# Analysis of the Dynamics of Clinical Parameters and Serum Angiogenic Factors in Systemic Sclerosis Patients Undergoing Tocilizumab Treatment

**DOI:** 10.1111/exd.70220

**Published:** 2026-02-09

**Authors:** Yuichiro Segawa, Takehiro Takahashi, Takuya Takahashi, Kenta Oka, Yumi Kambayashi, Toshiya Takahashi, Yoshihide Asano

**Affiliations:** ^1^ Department of Dermatology Tohoku University Graduate School of Medicine Sendai Japan

**Keywords:** interleukin 6, nailfold videocapillaroscopy, systemic sclerosis, tocilizumab, vasculopathy

## Abstract

Systemic sclerosis (SSc) is a systemic autoimmune disease characterised by vasculopathy and fibrosis of the skin and internal organs. In SSc, normal angiogenic processes are impaired due to abnormalities in vascular endothelial cells and pericytes. Nailfold videocapillaroscopy (NVC) is useful for evaluating microvascular injury in SSc. Tocilizumab (TCZ), an anti‐IL‐6 receptor antibody, has demonstrated efficacy in SSc‐associated interstitial lung disease (SSc‐ILD); however, its effects on vascular abnormalities in SSc remain poorly understood. We evaluated longitudinal changes in NVC findings in 13 SSc patients treated with monthly intravenous TCZ. Capillary density significantly increased at 6 months compared with baseline. This increase was positively and strongly correlated with improvements in pulmonary function test results. To further explore the correlation between NVC findings and angiogenic mediators, we quantified serum levels of seven pivotal angiogenic factors before and 6 months after TCZ initiation using a multiplex immunoassay. Among the angiogenic factors, serum levels of vascular endothelial growth factor (VEGF)‐A, platelet endothelial cell adhesion molecule (PECAM)‐1 and hepatocyte growth factor (HGF) were significantly intercorrelated and significantly decreased after 6 months of treatment. Notably, serum HGF levels showed the strongest correlation with capillary density and were also significantly correlated with modified Rodnan skin score. Furthermore, the decrease in serum VEGF‐A levels was robustly associated with improvements in pulmonary function test results. Our results collectively suggest that TCZ treatment is associated with changes in systemic vascular abnormalities and angiogenic factor profiles in SSc, which are critically involved in the pathophysiology of SSc‐associated interstitial lung disease.

## Graphical abstract

This study demonstrates that tocilizumab (TCZ) treatment promotes microvascular recovery in systemic sclerosis (SSc), as evidenced by increased capillary density and that this improvement in vasculopathy is closely associated with enhanced pulmonary function. Our findings suggest that modulation of pathogenic angiogenic factor networks involving HGF, PECAM‐1 and VEGF‐A may play a key role in mediating these therapeutic effects.

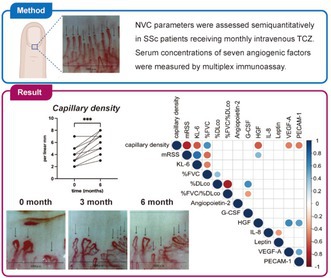

## 1 Introduction

Systemic sclerosis (SSc) is a systemic autoimmune disease characterised by vasculopathy and fibrosis of the skin and internal organs [1]. Interleukin‐6 (IL‐6) is a major regulator of the acute‐phase response and serum levels of IL‐6 are elevated in SSc patients [2]. The binding of IL‐6 to the IL‐6 receptor (IL‐6R) and the subsequent binding of this complex to gp130 receptor chains evoke signals, primarily through activation of the Janus kinase (JAK)/signal transducer and activator of transcription 3 (STAT3) pathway [3]. IL‐6 is primarily produced by myeloid cells upon Toll‐like receptor stimulation together with interleukin‐1β (IL‐1β) and tumour necrosis factor α (TNFα), which, via a feed‐forward loop, leads to an immense amplification of IL‐6 production during inflammatory conditions [4]. IL‐6, together with TNFα, promotes angiogenesis through phosphorylation of STAT3 and induction of vascular endothelial growth factor (VEGF)‐A production [5]. IL‐6 further induces the production of transforming growth factor‐β (TGF‐β), which plays a major role in the pathogenesis of fibrosis and it is also known to enhance TGF‐β signalling in fibroblasts [6].

In SSc, the normal angiogenic process is compromised due to abnormalities in vascular endothelial cells and pericytes. Vascular endothelial abnormalities also contribute to the fibrosis of SSc lesions through mechanisms such as endothelial‐to‐mesenchymal transition (EndoMT). Angiogenic mediators such as VEGF‐A are involved in vasculopathy due to the imbalance of angiogenic factors [7]. Microvascular changes in SSc can be observed as nailfold capillary abnormalities identified by nailfold videocapillaroscopy (NVC) [8]. Observation of nailfold capillary abnormalities is useful for the diagnosis of SSc and is included in the 2013 ACR/EULAR classification criteria for SSc [9]. Characteristic microvascular abnormalities include loss of capillary density, abnormal capillary dimensions and morphology and micro‐haemorrhages [10].

Tocilizumab (TCZ) is a monoclonal antibody targeting the IL‐6 receptor. TCZ has been shown to reduce serum VEGF levels in complete Freund's adjuvant (CFA)‐induced arthritic rats [11] and to decrease mean vessel density (MVD) in the synovium of rheumatoid arthritis (RA) patients, as evaluated by immunohistochemical staining for the endothelial marker CD31 [12]. However, the detailed mechanism by which TCZ inhibits aberrant angiogenesis is not yet fully understood. In 2021, TCZ became the first biologic medication approved for SSc‐associated interstitial lung disease (SSc‐ILD), based on the favourable results of the faSScinate (NCT01532869) and focuSSced (NCT02453256) trials [13, 14, 15].

Although TCZ has demonstrated efficacy in SSc‐ILD, no previous study has analysed its effect on longitudinal changes in capillaroscopic findings in SSc. In this study, we retrospectively evaluated NVC changes in SSc patients treated with TCZ. In addition, to clarify the correlation between temporal changes in NVC findings and angiogenic mediators involved in vascular abnormalities in SSc, we quantified serum levels of seven key angiogenic factors before and 6 months after TCZ initiation using a multiplex immunoassay.

## 2 Materials and Methods

### 2.1 Patients and Clinical Parameters

This retrospective study was conducted on an observational cohort of patients who visited Tohoku University Hospital between 1 April 2022 and 31 December 2023. A total of 13 Japanese patients with SSc were included (Table 1). Since TCZ has not yet been approved for the treatment of SSc or SSc‐ILD in Japan, its use was permitted under the approval of the Ethics Review Committee of Tohoku University Hospital. Disease duration was calculated from the first observed manifestation of SSc other than Raynaud's phenomenon. The diagnosis of SSc was based on the 2013 ACR/EULAR classification criteria for SSc [9]. Patients received intravenous TCZ (8 mg/kg body weight) every 4 weeks. Clinical information was retrospectively collected for all patients through a review of their medical records. The diagnosis of ILD was based on chest high‐resolution computed tomography findings. All participants provided written informed consent and the study protocol was approved by Tohoku University Hospital (No. 2022–1‐097).

**TABLE 1:** Baseline clinical and laboratory characteristics of the 13 SSc patients enrolled in the study.

Patient	1	2	3	4	5	6	7	8	9	10	11	12	13
Age	55	56	73	57	49	62	43	38	60	37	41	64	71
Sex	F	F	F	F	F	F	F	F	F	M	F	M	F
Duration (years)	2.5	0.7	1.5	32	36	17	2	7	4.3	7	4	1.3	3
ILD	+	+	+	+	+	+	+	+	+	+	+	+	+
mRSS	13	24	6	13	20	11	11	15	2	19	6	18	29
%FVC (%)	94.2	97.6	126.9	77.1	78	71.3	91.5	76.8	94.2	74.7	98.4	83.8	72.5
%DLco (%)	107	107.4	75.5	82.7	74.4	70.6	86.4	86.4	77.7	61.2	115	105	105.6
KL‐6 (U/mL)	588	756	311	758	947	515	563	160	892	349	182	343	3313
CRP (mg/mL)	1	0.02	0.08	0.05	0.2	0.39	0.03	0.01	0.05	0.15	0.02	0.02	0.05
ACA (index)	—	—	—	—	—	—	—	—	—	—	—	—	—
Topo‐I (index)	—	660	—	532	850	850	850	—	414	—	850	—	—
RNAPIII (index)	—	—	—	—	—	—	—	—	—	—	—	29.8	150
Prior treatment	—	—	—	Bosentan	Bosentan Ninte danib	MMF	PSL RTX	PSL	Bosentan Ninte danib PSL	—	—	—	—
NVC pattern	A	L	N	L	L	L	N	L	N	L	N	A	L

Abbreviations: A, active scleroderma pattern; ACA, anti‐centromere antibody; CRP, C‐reactive protein; DLco, diffusing capacity of the lung for carbon monoxide; F, female; FVC, forced vital capacity; ILD, interstitial lung disease; KL‐6, Krebs von den Lungen‐6; L, late scleroderma pattern; M, male; MMF, mycophenolate mofetil; mRSS, modified Rodnan skin score; N, mon‐scleroderma pattern; PSL, prednisolone; RNAPIII, anti‐RNA polymerase III antibody; RTX, rituximab; Topo‐I, anti‐topoisomerase I antibody.

### 2.2 Nailfold Capillaroscopy

We longitudinally assessed NVC findings in each patient using the GOKO Bscan/Bscan‐Z (GOKO Imaging Devices, Kawasaki, Japan) videocapillaroscopy system, coinciding with the timing of monthly TCZ administration. Each patient underwent examination with a standardised NVC technique [10]. The fourth digit of the right hand was examined. Immersion oil was applied to the nailfold just proximal to the nail to enhance skin transparency. Central images of each nailfold were captured and a grid corresponding to a 1 mm scale was placed on all images. Quantitative NVC assessment within the 1 mm grid included the following parameters at the image level: capillary density (number of capillaries in the distal row); capillary dimension (number of dilated capillaries [dilations] with an apical limb diameter of 20–50 μm and number of giant capillaries [giants] with an apical limb diameter > 50 μm); capillary morphology number of capillaries with normal morphology, such as hairpin shape, once‐ or twice‐crossing shape, or tortuous shape in which limbs bend but do not cross, with a convex tip and number of capillaries with abnormal morphology, defined as any morphology not meeting the criteria for normal [16, 17]; and micro‐haemorrhages (red or brown amorphous structures in the pericapillary or periungual region). The NVC pattern before initiation of TCZ treatment was evaluated according to previously reported criteria [10].

### 2.3 Assays for Serum Angiogenic Factors

Serum samples were collected before and 6 months after initiation of monthly TCZ administration and stored at −80°C until measurement. Serum levels of seven angiogenic factors, including angiopoietin‐2, granulocyte colony‐stimulating factor (G‐CSF), hepatocyte growth factor (HGF), interleukin‐8 (IL‐8), leptin, VEGF‐A, and platelet endothelial cell adhesion molecule‐1 (PECAM‐1), were measured using a Luminex Human Discovery Assay (R and D Systems, #LXSAHM‐07) according to the manufacturer's instructions on a Bio‐Plex 200 system (Bio‐Rad Laboratories, Hercules, CA, USA).

### 2.4 Statistical Analysis

Statistical analyses were performed using GraphPad Prism, version 10 (GraphPad Software, La Jolla, CA, USA). Nonparametric comparisons were performed using the Wilcoxon signed‐rank test. Correlations between serum angiogenic factors and clinical parameters were analysed by simple linear regression. Normality of continuous variables was assessed using the Shapiro–Wilk test. Spearman's rank correlation heatmaps were generated using R version 4.5.1. A *p*‐value < 0.05 was considered statistically significant.

## 3 Results

### 3.1 Changes in mRSS and Lung Function During TCZ Treatment in the Study Cohort

**FIGURE 1:**
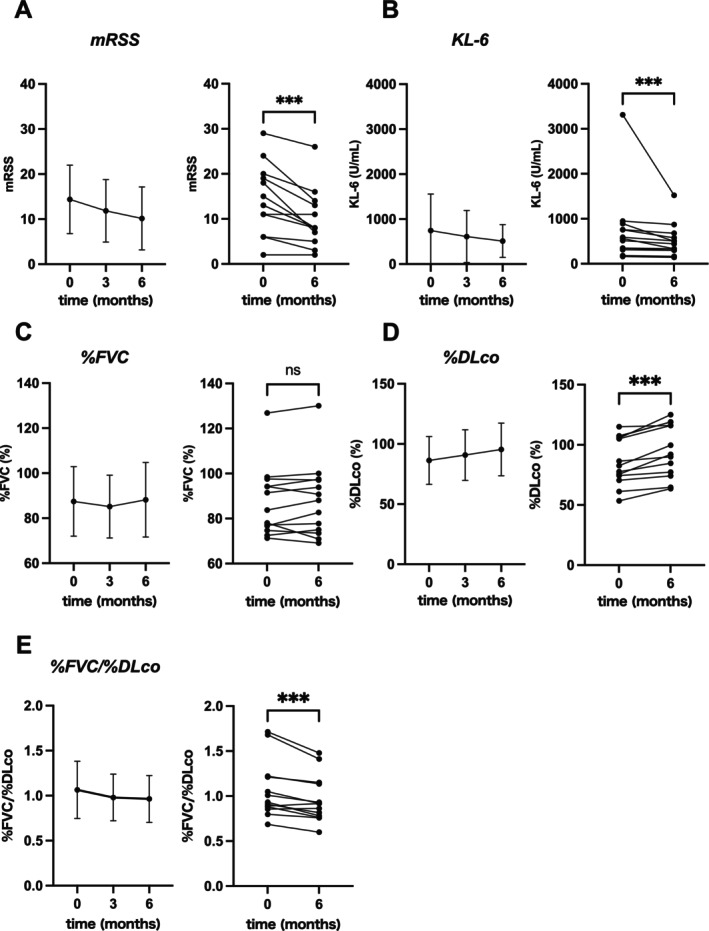
Changes in clinical parameters of SSc during TCZ treatment. Changes in each clinical parameter during TCZ treatment are shown in the following panels: (A) mRSS, (B) KL‐6, (C) %FVC, (D) %DLco and (E) %FVC/%DLco. Data are expressed as mean ± SD. Statistical significance is indicated as ***p < 0.001; NS, not significant (*p* < 0.05).

After the initiation of TCZ treatment, mRSS showed a decreasing trend in all cases, with a significant reduction compared to pre‐treatment scores at 6 months (*p* = 0.001) (Figure 1A). Similarly, serum KL‐6 levels significantly decreased at 6 months compared with baseline (*p* = 0.0002) (Figure 1B). Pulmonary function tests (PFTs) are crucial for long‐term monitoring of SSc‐ILD, with forced vital capacity (FVC) and diffusing capacity of the lungs for carbon monoxide (DLco) being key parameters for assessing lung function in SSc patients [18]. While %FVC showed no significant change after 6 months, %DLco significantly increased compared to pre‐treatment values (*p* = 0.0002) (Figure 1C,D). In addition, the %FVC/%DLco ratio also significantly increased (*p* = 0.001) (Figure 1E).

### 3.2 Longitudinal Observation of NVC Changes During TCZ Treatment

**FIGURE 2:**
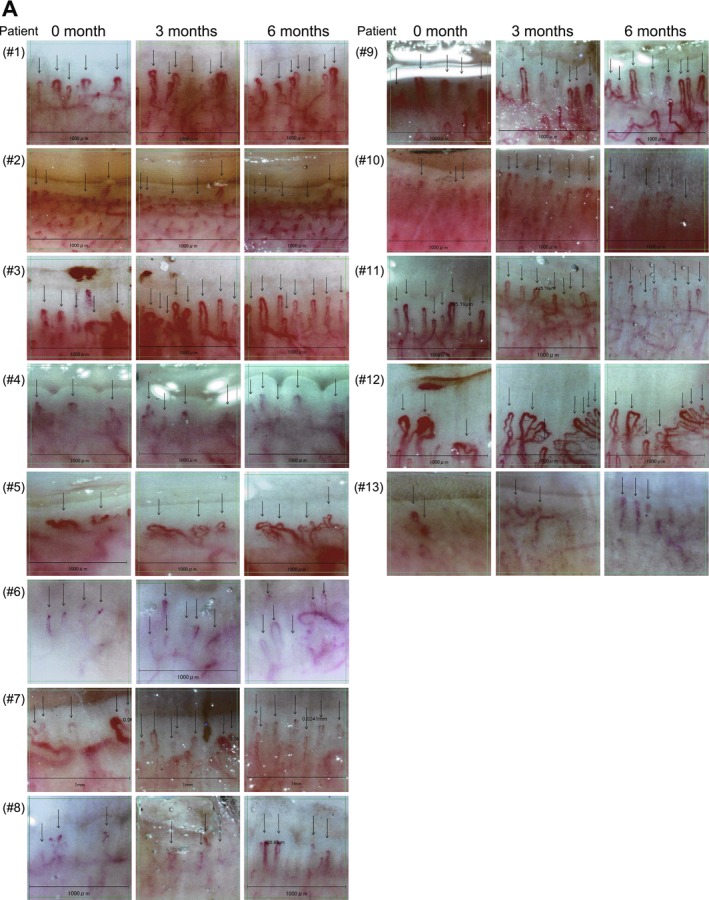
Longitudinal NVC images in 13 SSc patients. (A) NVC images obtained before and at 3 and 6 months after TCZ administration in 13 SSc patients. Patient numbers correspond to those in Table 2. Longitudinal NVC images were acquired along the midline of the right fourth digit.

**FIGURE 3:**
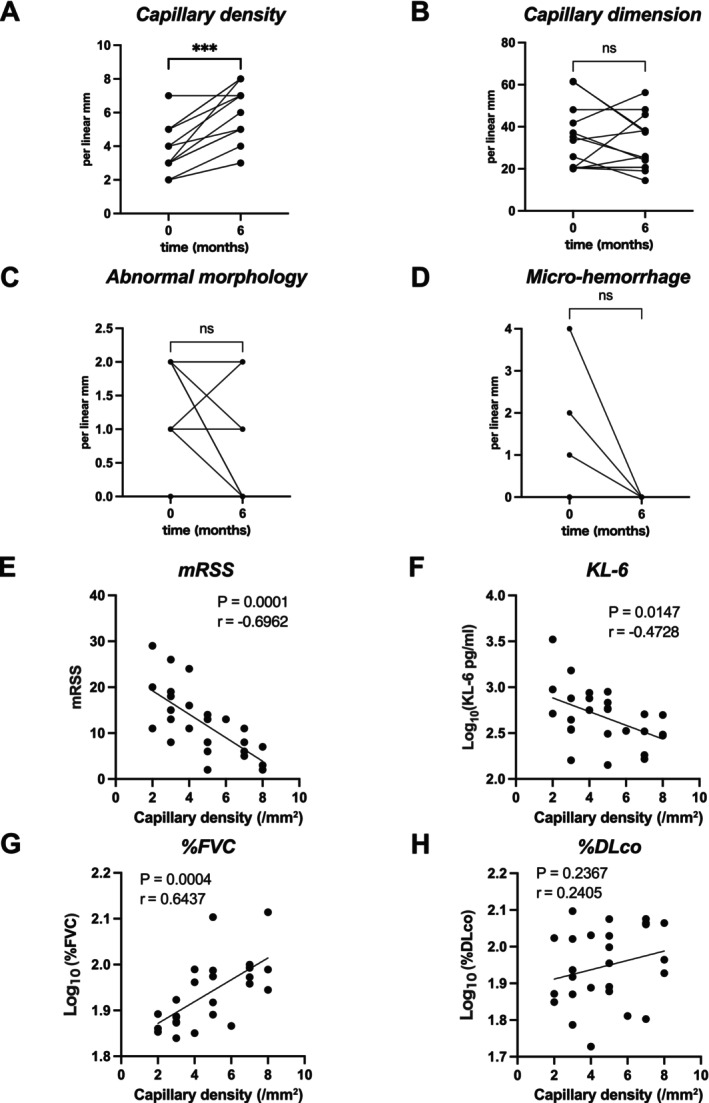
Changes in capillaroscopic parameters during TCZ treatment and correlations of capillary density with mRSS, KL‐6 and pulmonary function. Changes in NVC parameters after the initiation of TCZ treatment are summarised in the following panels. (A) Capillary density; (B) Capillary dimension; (C) Capillary morphology; (D) Micro‐haemorrhages. Correlations of capillary density with mRSS (E), KL‐6 (F), %FVC (G) and %DLco (H) are shown.

We longitudinally evaluated NVC images of 13 SSc patients at baseline, 3 months and 6 months after TCZ initiation, with representative images for each patient and time point shown in Figure 2A. Although variability was observed among patients, there was a notable trend toward restoration of normal capillary density and morphology over time. Before treatment, all patients exhibited avascular areas lacking normal capillary loops. However, by 3 months after TCZ initiation, normal hairpin‐shaped capillaries began to appear in these avascular areas (see Figure 2A, Patients #1, #3 and #12). In some cases, giant capillaries transformed into normal hairpin capillaries after TCZ treatment (see Figure 2A, Patient #12). To quantify these changes, we analysed NVC parameters longitudinally. Capillary density significantly increased at 6 months compared to baseline (Figure 3A; individual patient data in Table 2). Possibly due to the small sample size, no statistically significant differences were observed for capillary dimension or morphology (Figure 3B,C). Similarly, no statistically significant change in micro‐haemorrhage density was detected (Figure 3D), although micro‐haemorrhages were absent in all cases at 6 months.

**TABLE 2:** Changes in laboratory data and NVC findings before and 6 months after TCZ treatment.

Patient		1	2	3	4	5	6	7	8	9	10	11	12	13	0 month versus 6 months *p*‐value
mRSS	0 month	13	24	6	13	20	11	11	15	2	19	6	18	29	0.001***
	6 months	8	14	3	8	16	8	11	8	2	13	5	7	26	
%FVC (%)	0 month	94.2	97.6	127	77.1	78	71.3	91.5	76.8	94.2	74.7	98.4	83.8	72.5	0.38
	6 months	90.8	97.1	130	77.8	70.9	69.1	67.2	82.7	97.5	73.5	100	88.1	75	
%DLco (%)	0 month	107	107	75.5	82.7	74.4	70.6	19.2	86.4	77.7	61.2	115	105	106	0.0002***
	6 months	119	118	92.1	99.7	77.3	74.2	23.1	90.1	84.7	64.7	116	116	125	
KL‐6 (U/mL)	0 month	588	756	311	758	947	515	563	160	892	349	182	343	3313	0.0002***
	6 months	507	576	297	679	870	443	331	142	499	334	165	305	1520	
%FVC/%DLco	0 month	0.88	0.91	1.68	0.93	1.05	1.01	4.77	0.89	1.21	1.22	0.86	0.80	0.69	0.001***
	6 months	0.76	0.82	1.41	0.78	0.92	0.93	2.91	0.92	1.15	1.14	0.86	0.76	0.60	
Capillary density	0 month	5	4	5	3	2	4	4	3	5	3	7	3	2	0.0005***
	6 months	7	5	8	5	4	6	7	5	8	6	7	8	3	
Capillary dimension	0 month	61.5	20.7	41.7	33.5	20.3	20.4	37.1	19.9	48.1	20.4	35.1	61.3	25.7	0.68
	6 months	37.4	20.7	56.2	38.1	45.7	20.7	24.1	25.9	48.1	19	25.1	38.1	14.4	
Abnormal morphology	0 month	0	1	2	2	1	2	0	2	0	0	0	2	2	0.13
	6 months	0	2	0	2	2	0	0	1	0	0	0	2	0	
Micro‐haemorrhage	0 month	0	0	4	0	0	0	0	0	0	1	0	2	0	0.25
	6 months	0	0	0	0	0	0	0	0	0	0	0	0	0	

*Note:* Units for each NVC parameter are as follows: capillary density (/mm^2^), capillary dimension (/mm), capillary morphology (/mm^2^) and micro‐haemorrhages (/mm^2^). Nonparametric correlations were analysed using the Wilcoxon signed‐rank test. A *p*‐value < 0.05 was considered statistically significant.

*** *p* < 0.001.

### 3.3 Correlation Between Capillary Density and Clinical Parameters Before and After TCZ Treatment

Given the significant increase in capillary density at 6 months (Figure 3A), we examined its association with clinical parameters. Correlation analyses between capillary density and mRSS, KL‐6, %FVC and %DLco were performed at baseline and 6 months. While %DLco showed no significant correlation (Figure 3H), mRSS, KL‐6 and %FVC each demonstrated a significant, strong correlation with capillary density (*r* = −0.70, −0.47 and 0.64, respectively; all *p* < 0.05) (Figure 3E–G), suggesting that skin sclerosis and pulmonary function are closely associated with capillary abnormalities, particularly with capillary density in SSc.

### 3.4 Dynamics of Seven Key Serum Angiogenic Factors Before and After TCZ Treatment and Their Association With Capillary Density and Clinical Parameters

**FIGURE 4:**
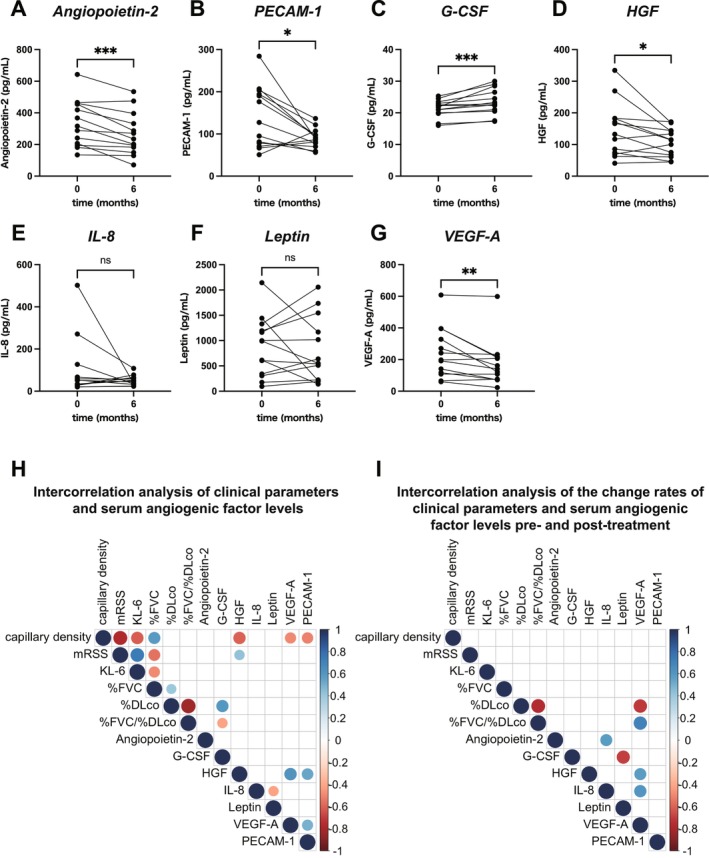
Dynamics of seven serum angiogenic factors and their correlations with mRSS, capillary density, KL‐6 and pulmonary function. Dynamics of seven serum angiogenic factors before and after TCZ treatment are summarised in the following panels: (A) Angiopoietin‐2; (B) PECAM‐1; (C) G‐CSF; (D) HGF; (E) IL‐8; (F) Leptin; (G) VEGF‐A. *p < 0.05; **p < 0.01; ***p < 0.001. (H) Spearman's rank correlation heatmaps of the indicated parameters are shown. For pulmonary function and serum angiogenic factors, log‐transformed values were used to calculate correlations. Values at both pre‐treatment and post‐treatment (6 months after initiation) were included in the calculations. (I) Spearman's rank correlation heatmaps showing the rate of change from baseline to 6 months after TCZ treatment. In (H) and (I), the size and colour of the circles indicate the Spearman's rank correlation coefficient (ρ) and only statistically significant correlations (*p* < 0.05) are displayed.

Given the established role of aberrant angiogenesis in SSc pathogenesis, we examined serum levels of seven angiogenic factors implicated in SSc, including angiopoietin‐2, PECAM‐1, G‐CSF, HGF, IL‐8, leptin and VEGF‐A, before and 6 months after TCZ initiation [19, 20, 21, 22, 23]. Serum levels of angiopoietin‐2, PECAM‐1, HGF and VEGF‐A significantly decreased after TCZ administration (Figure 4A,B,D,G), whereas G‐CSF significantly increased (Figure 4C). No significant changes were observed in IL‐8 or leptin levels (Figure 4E,F). To investigate the clinical relevance of these angiogenic changes, we performed Spearman's rank correlation analyses between each factor, capillary density and clinical parameters, including mRSS, KL‐6 and pulmonary function test results (%FVC, %DLco and %FVC/%DLco). As shown in the correlation heatmap (Figure 4H), HGF exhibited the strongest negative correlation with capillary density (Spearman's ρ = −0.61, *p* < 0.05). VEGF‐A and PECAM‐1 also showed significant negative correlations (Spearman's ρ = −0.50 and −0.51, respectively; both *p* < 0.05) and these three factors were intercorrelated. G‐CSF showed no significant correlation with capillary density, but was significantly correlated with %DLco and %FVC/%DLco (Spearman's ρ = 0.59 and −0.42, respectively; both *p* < 0.05).

Finally, we analysed inter‐correlations between change rates ([post‐treatment value—pre‐treatment value]/pre‐treatment value) of each parameter from baseline to 6 months using Spearman's rank correlation (Figure 4I). Notably, the change rate of VEGF‐A strongly correlated with change rates in %DLco and %FVC/%DLco (Spearman's ρ = −0.72 and 0.71, respectively, both *p* < 0.05).

## 4 Discussion

Previous studies have demonstrated that various soluble mediators, such as cytokines, growth factors, chemokines and soluble forms of cell adhesion molecules, are elevated in the sera of SSc patients [24, 25]. IL‐6 is a cytokine with pleiotropic activities, including stimulating the proliferation and differentiation of B and T lymphocytes, enhancing antibody production, activating T cells, stimulating haematopoietic precursors to differentiate, influencing the proliferative capacity of non‐lymphoid cells and activating the acute phase protein response [26]. Circulating levels of IL‐6 are elevated in patients with SSc and are associated with the development of skin fibrosis and SSc‐ILD [27, 28]. Serum IL‐6 levels in SSc patients have also been reported to correlate with mRSS [27]. In this study, a significant improvement in mRSS was observed after TCZ treatment (Figure 1A). However, it should be noted that this was not a prospective randomised study, nor was the evaluator blinded to the treatment. Moreover, in previous randomised controlled studies [13, 15], mRSS tended to decrease in the placebo‐treated group as well, as expected from the natural disease course.

Interstitial lung disease (ILD) is the internal organ involvement most critically associated with mortality in SSc [29]. Up to 90% of patients with SSc may exhibit radiological changes of ILD on high‐resolution CT (HRCT) of the thorax, whereas 40%–75% of patients may show pulmonary function test (PFT) abnormalities [30]. Both %FVC and %DLco are prognostic factors in patients with SSc‐ILD [31], with %DLco generally considered more sensitive to pulmonary involvement than %FVC [32]. In this study, significant improvement in %DLco and %FVC/%DLco was observed 6 months after TCZ treatment (Figure 1D,E). The %FVC/%DLco ratio has been suggested as a sensitive marker to detect early changes in pulmonary blood flow due to vascular impairment, with a threshold of 1.6 used to suspect pulmonary arterial hypertension (PAH) [33]. Our observations suggest that TCZ treatment may be associated with improvements in pulmonary function in SSc patients, at least in part in association with changes in pulmonary vascular abnormalities.

Our results revealed that capillary density was significantly correlated with KL‐6 and %FVC (Figure 3F,G). Of note, patient #12, who exhibited marked improvement in capillary density (Figure 2A), showed particularly remarkable increases in pulmonary function (Table 2). In contrast, patients with little change in capillary density had minimal change in pulmonary function (patient #2 and patient #11; see Figure 2A and Table 2). This suggests that improvement in capillary density is closely related to amelioration of pulmonary function in SSc patients and that the dynamics of capillary density could serve as an important biomarker for responsiveness to TCZ treatment.

In this study, four angiogenic factors, such as angiopoietin‐2, HGF, PECAM‐1 and VEGF‐A, were significantly decreased over the 6‐month course of TCZ treatment (Figure 4A,B,D,G). Furthermore, HGF, PECAM‐1 and VEGF‐A were intercorrelated and showed significant negative correlations with capillary density. HGF was initially thought to be a growth factor specific to liver cells, but has since been found to be involved in the proliferation and migration of vascular endothelial cells [34]. Serum HGF levels have been reported to correlate with the onset of pulmonary hypertension in SSc patients [35]. Although there are few reports on the pathological significance of HGF in SSc, our findings indicate that HGF strongly correlates with capillary density and mRSS, suggesting that it is deeply involved in vascular disorders preceding fibrosis in SSc. PECAM‐1 is expressed in leukocytes, platelets and endothelial cells and exerts its effects through the translocation of integrin α6β1 [36]. Elevated levels of PECAM‐1 have been suggested to be associated with vascular endothelial dysfunction, vascular damage and an increased risk of cardiovascular disease [36]. In this study, serum PECAM‐1 levels significantly decreased following TCZ treatment in patients with SSc and PECAM‐1 showed a strong correlation with capillary density (Figure 4B,H). These findings suggest that TCZ treatment may be associated with changes in vascular and endothelial damage and that PECAM‐1 may be involved in vascular injury in SSc. G‐CSF was the only factor to show a significant increase 6 months after TCZ treatment (Figure 4C). G‐CSF is a major regulator of neutrophil production and function [37] and promotes mobilisation of bone marrow mesenchymal stem cells (MSCs), which possess multiple regenerative and angiogenic properties, to the peripheral blood and tissues [38, 39]. Consistently, G‐CSF treatment has been reported to be useful for refractory digital ulcers in SSc patients [40]. Although the precise mechanisms remain to be elucidated, the observed increase in G‐CSF following TCZ treatment may be associated with enhanced MSC mobilisation and could be linked to vascular and tissue repair processes.

One of the features associated with vascular injury in SSc is elevated serum VEGF‐A, a major angiogenic mediator [41]. Although VEGF‐A essentially promotes proliferation of endothelial cells and pericytes to repair injured vessels, previous studies have revealed that persistent upregulation of VEGF‐A over a long period can cause the vascular network to become disorganised, eventually leading to vascular degeneration and loss [42]. We observed a remarkable emergence of new, well‐organised, hairpin‐shaped capillary loops over time after TCZ initiation, even in previously avascular areas. This was reflected in the significant increase in capillary density after treatment, which was significantly coupled with a decrease in serum VEGF‐A levels. Of note, the serum levels of G‐CSF as well as the change rate of VEGF‐A showed significant correlations with the values or the change rates of %DLCO and %FVC/%DLCO, respectively (Figure 4H,I). These parameters have previously been reported to strongly reflect vascular involvement in SSc‐ILD [43, 44]. Collectively, these findings suggest that inhibition of IL‐6/IL‐6 receptor signalling by TCZ may be associated with suppression of persistent overproduction of VEGF‐A and with changes in vascular features, possibly in relation to increased G‐CSF levels and MSC recruitment, which may be linked to partial modification of pathological vascular abnormalities in SSc patients.

There are several limitations to the present study. It was performed retrospectively; thus, a prospective study is needed to confirm the results. Additionally, this study had a limited sample size and included only Japanese patients, which limits generalisability of the findings. There is wide variation in disease duration among SSc patients and treatment prior to TCZ was not standardised. Furthermore, potential influences such as temperature or other seasonal effects, which were not assessed in this study, may have affected the results. Regarding the measurement of serum angiogenic factors, we did not have samples from age‐ and sex‐matched control individuals, precluding comparison with healthy controls. Future studies that address these issues and include larger numbers of patients from different ethnicities will be necessary to confirm our findings.

In conclusion, our retrospective evaluation of longitudinal changes in NVC findings in SSc patients undergoing TCZ treatment revealed a significant and sustained increase in capillary density over the course of treatment. Changes in capillary density, or restoration of lost capillaries, were significantly correlated with improvements in pulmonary function. Serum HGF, PECAM‐1 and VEGF‐A levels were significantly correlated with capillary density and TCZ treatment resulted in a significant decrease in these factors. Overall, our findings indicate that TCZ treatment is accompanied by changes in systemic vascular and capillaroscopic features in SSc, although further studies are needed to clarify the underlying mechanisms.

## Author Contributions

Yuichiro Segawa collected and analysed nailfold capillaroscopy images, performed multiplex immunoassays and drafted the manuscript. Takehiro Takahashi, Takuya Takahashi, Kenta Oka, Yumi Kambayashi and Toshiya Takahashi collected serum samples as well as clinical and laboratory data. Takehiro Takahashi and Yoshihide Asano critically revised the manuscript. All authors read and approved the final version of the manuscript.

## Funding

This work was supported by the Ministry of Education, Culture, Sports, Science and Technology (MEXT) KAKENHI [22K21370 and 23K24354].

## Ethics Statement

Reviewed and approved by Tohoku University Hospital; approval #2022–1‐097.

## Consent

The patients in this manuscript have given written informed consent for the publication of their case details.

## Conflicts of Interest

The authors declare no conflicts of interest.

## Data Availability

The data that support the findings of this study are available from the corresponding author upon reasonable request.
